# Supplementary material for: From sequence to enzyme mechanism using multi-label machine learning
Source: BMC Bioinformatics. 2014 May 19;15:150. doi: 10.1186/1471-2105-15-150 (PMC4229970; doi:10.1186/1471-2105-15-150)
Supplement: Additional file 2 — Java code of ml2db. Additional file ml2db_code.tar.gz contains the Java source code to run the multi-label machine learning experiments and save the results to database. The code’s Javadoc is included. [file 1471-2105-15-150-S2.zip › additional file 2/ml2db/utils/doc/uk/ac/standrews/utils/main/database/TableRow.html]

TableRow


JavaScript is disabled on your browser.


- Overview
- Package
- Class
- Use
- Tree
- Deprecated
- Index
- Help

- Prev Class
- Next Class

- Frames
- No Frames

- All Classes

- Summary:
- Nested |
- Field |
- Constr |
- Method

- Detail:
- Field |
- Constr |
- Method


uk.ac.standrews.utils.main.database

## Class TableRow

- java.lang.Object
- - uk.ac.standrews.utils.main.database.Manager
  - - uk.ac.standrews.utils.main.database.DbManager
    - - uk.ac.standrews.utils.main.database.TableManager
      - - uk.ac.standrews.utils.main.database.TableRow

- All Implemented Interfaces:
  :   java.io.Serializable

  ---

    

  ```
  public class TableRow
  extends TableManager
  ```

  Contains the data for a generic row (a list of [table column, value] entries)
  to be written to a database table.

  Version:
  :   9 Jun 2010

  Author:
  :   Luna De Ferrari luna.deferrari-at-ed.ac.uk

  See Also:
  :   Serialized Form

- - ### Field Summary

    - ### Fields inherited from class uk.ac.standrews.utils.main.database.DbManager

      `m_dbWriter`
  - ### Constructor Summary

    Constructors

    Constructor and Description

    `TableRow(Table table)`

    `TableRow(Table table,
    java.util.TreeMap<java.lang.String,java.lang.String> columnToValueMap)`

    `TableRow(Table table,
    java.util.Vector<java.lang.String> columns,
    java.util.Vector<java.lang.String> values)`
  - ### Method Summary

    Methods

    | Modifier and Type | Method and Description |
    | --- | --- |
    | `boolean` | `checkRow()` |
    | `boolean` | `checkRowFields()` Checks that the record values are paired to fields existing in the table. |
    | `boolean` | `checkRowValues()` TODO Not implemented: check that each row value is of the appropriate datatype for the table field... |
    | `java.util.TreeMap<java.lang.String,java.lang.String>` | `getColumn2ValueMap()` Returns a comma separated string of the values |
    | `java.util.Vector<java.lang.String>` | `getColumns()` |
    | `java.lang.String` | `getValue(java.lang.String column)` Return the value for a certain column |
    | `java.util.Vector<java.lang.String>` | `getValues()` |
    | `boolean` | `isCorrect()` |
    | `void` | `setColumns2Values(java.util.TreeMap<java.lang.String,java.lang.String> map)` |
    | `java.lang.String` | `toString()` Returns a comma separated string of the values |

    - ### Methods inherited from class uk.ac.standrews.utils.main.database.TableManager

      `getTable, getTableName`
    - ### Methods inherited from class uk.ac.standrews.utils.main.database.DbManager

      `closeConnection, connectionIsValid, getDatabaseName, getDbConnection, getDbCreator, getDbReader, getDbWriter, refreshConnection, runAnalyzeTable`
    - ### Methods inherited from class java.lang.Object

      `equals, getClass, hashCode, notify, notifyAll, wait, wait, wait`

- - ### Constructor Detail


    - #### TableRow

      ```
      public TableRow(Table table)
      ```


    - #### TableRow

      ```
      public TableRow(Table table,
              java.util.TreeMap<java.lang.String,java.lang.String> columnToValueMap)
      ```


    - #### TableRow

      ```
      public TableRow(Table table,
              java.util.Vector<java.lang.String> columns,
              java.util.Vector<java.lang.String> values)
      ```
  - ### Method Detail


    - #### checkRow

      ```
      public boolean checkRow()
      ```


    - #### checkRowFields

      ```
      public boolean checkRowFields()
      ```

      Checks that the record values are paired to fields existing in the table.
      Worth doing? or better to let the sql insert into fail...

      Returns:
      :   true if the record values fields exist in the table


    - #### checkRowValues

      ```
      public boolean checkRowValues()
      ```

      TODO Not implemented: check that each row value is of the appropriate
      datatype for the table field...

      Returns:
      :   true if row values are of appropriate type for the corresponding
          field


    - #### getColumn2ValueMap

      ```
      public java.util.TreeMap<java.lang.String,java.lang.String> getColumn2ValueMap()
      ```

      Returns a comma separated string of the values

      Returns:
      :   a comma separated string of the values
          public String columnsToString() {
          return DbUtils.getListOfValuesString(this.getColumns(),
          DbUtils.SINGLE\_QUOTE\_CHARACTER) + "\n";
          }


    - #### getColumns

      ```
      public java.util.Vector<java.lang.String> getColumns()
      ```


    - #### getValue

      ```
      public java.lang.String getValue(java.lang.String column)
      ```

      Return the value for a certain column


    - #### getValues

      ```
      public java.util.Vector<java.lang.String> getValues()
      ```


    - #### isCorrect

      ```
      public boolean isCorrect()
      ```


    - #### setColumns2Values

      ```
      public void setColumns2Values(java.util.TreeMap<java.lang.String,java.lang.String> map)
      ```

      Parameters:
      :   `m_map` -


    - #### toString

      ```
      public java.lang.String toString()
      ```

      Returns a comma separated string of the values

      **Overrides:**
      :   `toString` in class `java.lang.Object`

      Returns:
      :   a comma separated string of the values


- Overview
- Package
- Class
- Use
- Tree
- Deprecated
- Index
- Help

- Prev Class
- Next Class

- Frames
- No Frames

- All Classes

- Summary:
- Nested |
- Field |
- Constr |
- Method

- Detail:
- Field |
- Constr |
- Method
